# Advances in Non-Small Cell Lung Cancer: Current Insights and Future Directions

**DOI:** 10.3390/jcm13144189

**Published:** 2024-07-18

**Authors:** Pankaj Garg, Sulabh Singhal, Prakash Kulkarni, David Horne, Jyoti Malhotra, Ravi Salgia, Sharad S. Singhal

**Affiliations:** 1 Department of Chemistry, GLA University, Mathura 281406, Uttar Pradesh, India; 2 Department of Internal Medicine, Drexel University College of Medicine, Philadelphia, PA 19104, USA; 3 Departments of Medical Oncology & Therapeutics Research, Beckman Research Institute of City of Hope, Comprehensive Cancer Center and National Medical Center, Duarte, CA 91010, USA; 4 Departments of Molecular Medicine, Beckman Research Institute of City of Hope, Comprehensive Cancer Center and National Medical Center, Duarte, CA 91010, USA

**Keywords:** non-small cell lung cancer (NSCLC), lung cancer diagnosis, targeted therapies, genetic profiling, therapeutic resistance, CAR-T cell therapy

## Abstract

The leading cause of cancer deaths worldwide is attributed to non-small cell lung cancer (NSCLC), necessitating a continual focus on improving the diagnosis and treatment of this disease. In this review, the latest breakthroughs and emerging trends in managing NSCLC are highlighted. Major advancements in diagnostic methods, including better imaging technologies and the utilization of molecular biomarkers, are discussed. These advancements have greatly enhanced early detection and personalized treatment plans. Significant improvements in patient outcomes have been achieved by new targeted therapies and immunotherapies, providing new hope for individuals with advanced NSCLC. This review discusses the persistent challenges in accessing advanced treatments and their associated costs despite recent progress. Promising research into new therapies, such as CAR-T cell therapy and oncolytic viruses, which could further revolutionize NSCLC treatment, is also highlighted. This review aims to inform and inspire continued efforts to improve outcomes for NSCLC patients globally, by offering a comprehensive overview of the current state of NSCLC treatment and future possibilities.

## 1. Introduction

Non-small cell lung cancer (NSCLC) is a significant global health issue because it occurs frequently and has a high death rate. It makes up about 85% of all lung cancer cases, making it the most common form of lung cancer [1]. Recent World Health Organization (WHO) statistics indicate that lung cancer is the leading cause of cancer-related deaths worldwide, with around 1.8 million people dying from it each year [2]. The occurrence of NSCLC varies around the world due to differences in smoking habits, environmental factors, and healthcare quality. In developed countries, NSCLC cases are slowly decreasing thanks to successful anti-smoking campaigns and laws [3]. However, in developing countries, NSCLC cases are rising due to higher smoking rates and growing industrialization. Smoking is the main cause of NSCLC, accounting for about 85% of lung cancer cases. Tobacco smoke contains many carcinogens that damage DNA, causing genetic mutations and cancer. The risk of NSCLC is closely linked to how long and how much a person smokes, and it remains high even years after quitting. Additionally, exposure to secondhand smoke significantly raises the risk of lung cancer in non-smokers [4].

**Figure 1:**
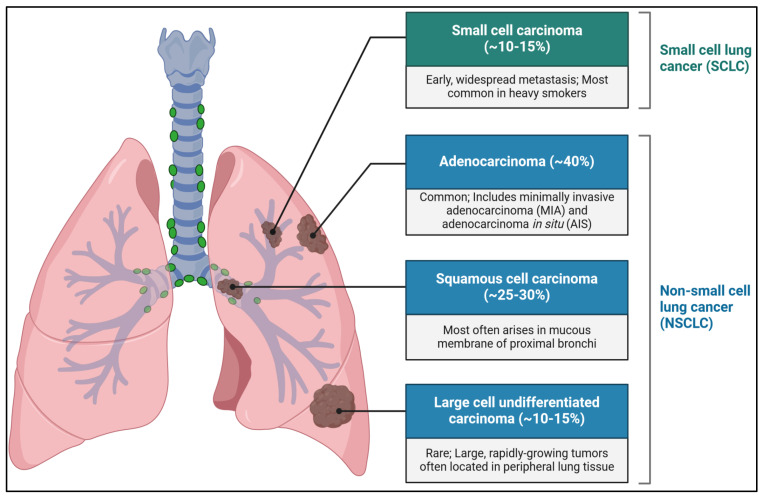
Overview of NSCLC types of cancers that develop from the lung’s epithelial cells, with three main subtypes: adenocarcinoma, squamous cell carcinoma, and large cell carcinoma.

Environmental factors are also a major risk factor for NSCLC. Exposure to harmful substances at work, such as asbestos, arsenic, chromium, and diesel exhaust, increases the risk of lung cancer [5]. Radon gas, a naturally occurring radioactive gas found in soil and building materials, is the second leading cause of lung cancer after smoking. Air pollution, particularly fine particulate matter (PM2.5), is also linked to higher rates of lung cancer, especially in urban areas with heavy traffic and industrial activity [6]. Genetic factors significantly impact the likelihood of developing NSCLC. Having a family history of lung cancer increases the risk, indicating a hereditary aspect. Certain genetic variations and mutations, like those in the p53 tumor suppressor gene, are associated with a higher risk of NSCLC. Additionally, specific mutations such as EGFR, KRAS, and ALK rearrangements not only affect the development of NSCLC but also serve as targets for personalized treatments [7]. NSCLC includes various types of cancers that develop from the lung’s epithelial cells, with three main subtypes: adenocarcinoma, squamous cell carcinoma, and large cell carcinoma (Figure 1). Each subtype has unique features that help in diagnosing and treating the NSCLC complex [8]. Adenocarcinoma is the most common and often occurs in the outer parts of the lung, frequently affecting non-smokers, and younger people. Squamous cell carcinoma usually develops in the central parts of the lungs and is closely linked to smoking. Large cell carcinoma is rarer, more aggressive, and tends to spread quickly [9]. The development of NSCLC involves several genetic mutations and changes in cell growth, differentiation, and death pathways. Common mutations in NSCLC include changes in the EGFR, ALK, ROS1, and KRAS genes, which promote the uncontrolled growth and spread of cancer cells [10]. Understanding these molecular mechanisms is crucial for creating targeted treatments that block these pathways and improve patient outcomes [11]. NSCLC is a major health issue because it often has a high death rate due to late diagnosis.

Early-stage NSCLC usually shows no symptoms or has vague symptoms like a persistent cough, chest pain, shortness of breath, and unexplained weight loss, which can delay diagnosis. Diagnostic tools such as chest X-rays, CT scans, and PET scans are vital for detecting and determining the stage of the disease [12]. Biopsy and histopathological examination are essential for confirming the diagnosis and identifying the subtype. Ongoing research aims to identify genetic risk factors for lung cancer to find high-risk individuals and develop preventive strategies [13]. The treatment for NSCLC has changed a lot in the past decade. Traditional treatments like surgery, radiation, and chemotherapy are still important for managing the disease. However, new targeted therapies and immunotherapies have significantly improved NSCLC treatment [14].

Targeted therapies, such as tyrosine kinase inhibitors (TKIs) for specific genetic mutations like EGFR and ALK, offer a personalized approach that boosts survival and quality of life. Immunotherapies, including immune checkpoint inhibitors like pembrolizumab and nivolumab, have proven effective in strengthening the body’s immune response against cancer cells, resulting in lasting benefits for some patients [15]. Despite these advances, there are still challenges in treating NSCLC. Issues such as treatment resistance, the diversity of the disease, and limited access to advanced therapies remain significant hurdles [16]. Research is ongoing to better understand resistance mechanisms, find new treatment targets, and develop combination therapies to address these challenges. Additionally, early detection through better screening methods, such as low-dose CT scans for high-risk individuals, and the use of molecular diagnostics like liquid biopsies, are crucial for improving patient outcomes and survival rates [17].

## 2. Mechanisms Underlying NSCLC Development

**Figure 2:**
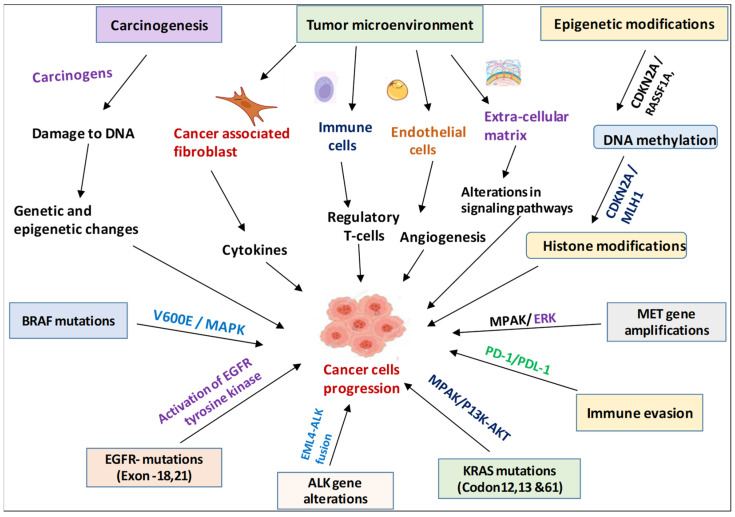
Causal determinants for the possible mechanism of NSCLC development.

The development of NSCLC is driven by a complex mix of genetic mutations, epigenetic changes, and interactions with the tumor’s surrounding environment (Figure 2). Understanding these mechanisms is crucial for creating targeted treatments and improving patient outcomes [18]. Advances in genetic profiling and molecular diagnostics have enabled personalized treatment approaches, significantly changing how NSCLC is managed. Ongoing research continues to discover new genetic and molecular targets, offering hope for more effective treatments and better outcomes for patients with NSCLC [19]. NSCLC develops through a combination of genetic, environmental, and cellular factors. It starts with the accumulation of genetic mutations and epigenetic changes in the lung’s epithelial cells. These mutations interfere with normal cellular functions like cell cycle regulation, programmed cell death, and DNA repair, leading to uncontrolled cell growth and tumor development [20].

### 2.1. Carcinogenesis

The development of NSCLC often begins with exposure to carcinogens like tobacco smoke, asbestos, radon, and air pollutants. These substances cause direct damage to DNA, leading to mutations in key genes that regulate cell growth and division. The affected cells then start to multiply, driven by further genetic and epigenetic changes. This stage involves mutated cells growing more rapidly than normal cells. As more mutations and changes accumulate, the cells progress to form malignant tumors. At this point, they gain abilities like continuous blood vessel growth, avoiding cell death, invading tissues, and spreading to other parts of the body [21]. Chronic inflammation caused by exposure to carcinogens can create an environment that promotes tumor growth, leading to more genetic instability and mutations. Over time, these cancerous cells can evade the immune system, allowing them to grow unchecked. The interaction between genetic predispositions and environmental factors also affects how NSCLC develops differently in individuals. As the tumor progresses, it becomes more varied, making treatment more challenging and highlighting the need for personalized therapies [22]. Understanding the multi-step process of NSCLC development is crucial for creating prevention strategies, early detection methods, and effective treatments that can target cancer at different stages.

### 2.2. Tumor Microenvironment

The role of the tumor microenvironment is crucial in the development of NSCLC. It includes various cell types like cancer-associated fibroblasts, immune cells, endothelial cells, and components of the extracellular matrix. Interactions between cancer cells and these surrounding cells support tumor growth, new blood vessel formation, immune system evasion, and spread to other areas [23]. Cancer-associated fibroblasts release growth factors and cytokines that help cancer cells grow and invade tissues. Immune cells such as tumor-associated macrophages and regulatory T cells suppress the immune response and promote tumor growth, while endothelial cells promote angiogenesis, supplying the tumor with nutrients and oxygen. The extracellular matrix provides structural support and affects signaling pathways that drive tumor progression. Low oxygen conditions within the tumor environment are selected for more aggressive cancer cells, helping them evade the immune system [24]. It is important to understand these complex interactions for therapeutic strategies to be developed that target the tumor microenvironment, potentially disrupting the supportive niche that tumors need to grow and spread. Progress in this area is resulting in new therapies being developed that modify the tumor microenvironment, improving current treatments and outcomes for patients [25].

### 2.3. Genetic and Molecular Alterations in NSCLC

NSCLC is defined by many genetic and molecular changes that cause its development. These changes impact different pathways within cells that control growth, survival, and how cells mature. Common mutations in NSCLC involve genes like EGFR, KRAS, and ALK, which are crucial for cell communication and growth. There are also changes in genes like PIK3CA, BRAF, and MET that add to the formation and progression of tumors [26]. Epigenetic changes, like DNA methylation and histone modifications, also control how genes are expressed and how tumors behave. The TP53 and RB1 tumor suppressor genes are commonly turned off, which lets cells grow uncontrollably and causes genetic instability [27]. Advances in next-generation sequencing and other molecular diagnostic techniques have enabled the identification of these alterations, allowing for personalized treatment strategies that target specific genetic abnormalities [28]. Thus, knowing the intricate genetic makeup of NSCLC is vital for creating new targeted therapies and making better outcomes for patients.

#### 2.3.1. Epidermal Growth Factor Receptor (EGFR)

A notable portion of NSCLC, especially in adenocarcinoma and non-smokers, has activating mutations in the EGFR gene. These mutations are most common in specific parts (exons 18–21) of the EGFR gene, causing continuous activation of EGFR tyrosine kinase [29]. This leads to uncontrolled cell growth and survival. To counter this, targeted therapies like erlotinib and gefitinib, known as tyrosine kinase inhibitors (TKIs), have been created to block EGFR activity. These treatments have proven highly effective in patients with NSCLC that have EGFR mutations [30].

#### 2.3.2. Anaplastic Lymphoma Kinase (ALK)

A small percentage of NSCLC cases have rearrangements in the ALK gene, like the EML4-ALK fusion, mainly seen in non-smokers and younger patients. These rearrangements create an ALK tyrosine kinase that is always active [31]. This constant activity triggers pathways that drive cell growth and survival. ALK inhibitors such as crizotinib and alectinib have shown significant benefits for patients with NSCLC that have ALK rearrangements [32].

#### 2.3.3. KRAS

KRAS mutations are quite common in NSCLC, especially in adenocarcinomas and smokers. These mutations often occur in specific parts (codons 12, 13, and 61) of the KRAS gene. They activate pathways like MAPK and PI3K-AKT, promoting cell growth, survival, and resistance to cell death [33]. Developing effective targeted therapies for KRAS mutations has been challenging, but progress has been made with KRAS G^12^C inhibitors like sotorasib and adagrasib, showing promise in clinical trials and providing new hope for patients with this mutation [34].

#### 2.3.4. Other Genetic Alterations

##### ROS1

Rearrangements in the ROS1 gene, like those in ALK, create fusion proteins that are always active and promote cancerous signaling. Treating ROS1-rearranged NSCLC with ROS1 inhibitors like crizotinib has been effective. Newer ROS1 inhibitors such as entrectinib and lorlatinib are also in development to tackle resistance mechanisms and enhance outcomes for patients [35].

##### BRAF

BRAF mutations, especially V^600^E are present in a subset of NSCLC cases. Drugs called BRAF inhibitors, such as vemurafenib and dabrafenib, target these mutations and have proven effective in treating BRAF-mutant NSCLC. Combining BRAF inhibitors with MEK inhibitors, like trametinib, has also improved treatment outcomes by blocking multiple points in the signaling pathway [36].

##### MET

MET gene amplifications and exon 14 skipping mutations play a role in the development of NSCLC by activating the MET receptor tyrosine kinase. Drugs known as MET inhibitors, such as crizotinib and capmatinib, are used to target these changes. Newer treatments like tepotinib have shown significant benefits in clinical trials and are now being included in treatment plans for patients with MET-driven NSCLC [37].

#### 2.3.5. Epigenetic Changes

##### DNA Methylation

Unusual patterns of DNA methylation, like excessive methylation of gene promoters that control cell cycle and cell death, can silence important genes. These changes can be spotted using liquid biopsies, which could help diagnose the disease early and track how it progresses over time [38].

##### Histone Modifications

Alterations in histone acetylation and methylation pattern can affect chromatin structure and gene expression, promoting oncogenesis [39]. This can contribute to the development of cancer. Scientists are actively studying ways to target these changes using drugs like histone deacetylase (HDAC) inhibitors and other epigenetic drugs [40]. This research shows promise in reversing abnormal gene expression patterns in NSCLC.

#### 2.3.6. Immune Evasion

##### PD-1/PD-L1 Pathway

In NSCLC, cancer cells often use immune checkpoint pathways, like the PD-1/PD-L1 pathway, to avoid being detected and destroyed by the immune system. Immune checkpoint inhibitors such as pembrolizumab and nivolumab work by blocking these interactions, allowing the immune system to recognize and attack cancer cells again [41]. Combining checkpoint inhibitors with other treatments such as chemotherapy and targeted therapies has proven to be effective and is now a common approach in treating advanced NSCLC. Moreover, researchers are studying new checkpoint inhibitors and combination strategies to further improve outcomes for patients and expand the number of cases that can be successfully treated [42].

## 3. Cutting-Edge Imaging Techniques and Molecular Diagnostic Biomarkers for Pioneering NSCLC Diagnosis

**Figure 3:**
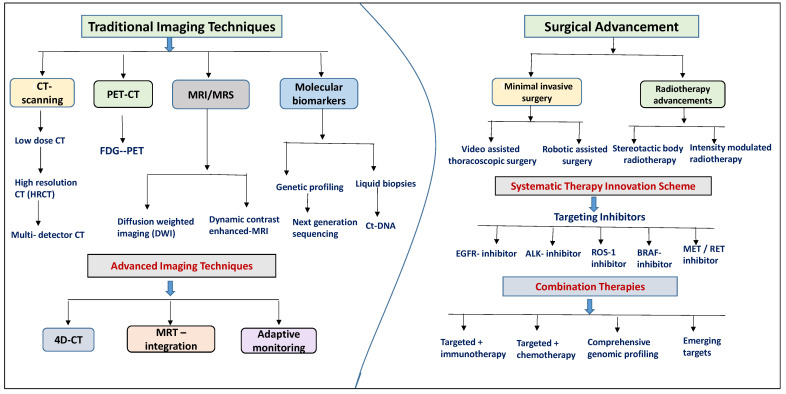
Systematic representation of all advanced techniques for innovative diagnosis and therapy interventions of NSCLC.

Getting an accurate and early diagnosis of NSCLC is crucial for effective treatment and better outcomes for patients. Advanced imaging technologies have greatly improved our ability to detect, assess the stage of, and monitor NSCLC over time. Among these technologies are computed tomography (CT), positron emission tomography–computed tomography (PET-CT), and magnetic resonance imaging (MRI), which have all seen significant advancements [43]. These improvements have made diagnostics more precise and less invasive. Traditional imaging methods such as CT, PET-CT, and MRI provide detailed information about the structure and function of tissues. Meanwhile, molecular diagnostics like genetic profiling and liquid biopsies allow for a precise understanding of the tumor at the molecular level (Figure 3). These advancements in technology enable us to detect NSCLC early, accurately determine its stage, and create personalized treatment plans. Ultimately, this leads to much better outcomes for patients [44].

### 3.1. Computed Tomography (CT)

CT scans play a crucial role in diagnosing and staging NSCLC. They provide detailed images of the chest in cross-sections, allowing doctors to spot lung nodules and masses accurately [45]. Recent advancements in CT technology have further improved its ability to diagnose.

#### 3.1.1. Low-Dose CT (LDCT)

LDCT has transformed lung cancer screening, especially for people at high risk like long-term smokers. It significantly lowers radiation exposure while still being highly sensitive in detecting early-stage lung cancers. Large studies like the National Lung Screening Trial (NLST) have shown that LDCT screening reduces lung cancer deaths by catching the disease earlier compared to regular chest X-rays [46].

#### 3.1.2. High-Resolution CT (HRCT)

HRCT provides even clearer images, making it easier to see lung tissue in detail and identify subtle abnormalities that might be missed on standard CT scans. It is particularly useful for understanding lung nodules and distinguishing between benign and malignant growths based on their features like edges, density, and growth patterns [47].

#### 3.1.3. Multi-Detector CT (MDCT)

MDCT machines use multiple detectors to quickly capture images covering larger areas of the body in one breath-hold. This reduces blurring due to movement and enhances image quality. MDCT is crucial for creating detailed maps of anatomy, essential for planning surgeries and radiotherapy treatments [48].

### 3.2. Positron Emission Tomography–Computed Tomography (PET-CT)

PET-CT combines the metabolic imaging of PET with the anatomical imaging of CT, making it a powerful tool for diagnosing, staging, and monitoring NSCLC. The most commonly used PET tracer, fluorodeoxyglucose (FDG-PET), highlights areas of high glucose metabolism, indicating cancerous activity [49]. This allows doctors to detect active tumors even before they show up on a CT scan. PET-CT is highly effective at detecting both primary tumors and metastatic disease, helping with accurate staging and treatment planning. It is better than CT alone for assessing lymph node involvement and distant metastasis, which are crucial for determining the right treatment strategy. PET-CT also helps evaluate the effectiveness of therapy by measuring changes in metabolic activity, providing early signs of whether a treatment is working or not. Research into new PET tracers that target specific molecular pathways, such as somatostatin receptors and amino acid transporters, is expanding the usefulness of PET-CT. These advancements allow for a more precise understanding of tumor biology and potential therapeutic targets [50].

### 3.3. Magnetic Resonance Imaging (MRI)

MRI provides superior soft tissue contrast compared to CT and PET-CT, making it an essential tool in the diagnosis and management of NSCLC. MRI is particularly effective at imaging soft tissues, blood vessels, and the brain, which is useful for evaluating chest wall involvement, mediastinal structures, and brain metastases [51]. Advanced MRI techniques like diffusion-weighted imaging (DWI) and dynamic contrast-enhanced MRI (DCE-MRI) offer detailed insights into tumor cellularity and vascularity. These techniques help distinguish malignant from benign lesions. Whole-body MRI is becoming a valuable non-invasive method for systemic staging, offering high sensitivity for detecting bone and liver metastases, which are common in advanced NSCLC.

Bone metastasis is a common complication in advanced NSCLC, occurring when cancer cells spread from the lungs to the bones. This condition can lead to severe pain, fractures, spinal cord compression, and hypercalcemia, significantly impacting the patient’s quality of life and prognosis. The treatment of bone metastasis in NSCLC is a multifaceted challenge requiring a comprehensive approach that includes pain management, prevention of skeletal related events (SREs), and innovative therapeutic strategies. Ongoing research and clinical trials hold promise for improving outcomes and quality of life for patients with bone metastases from NSCLC. Emerging therapies focus on targeted treatments for bone specific biology combination therapies to enhance efficacy, new bone-modifying agents, and the potential of immunotherapies. Additionally, MRI avoids ionizing radiation, making it a safer option for long-term monitoring and follow-up, particularly for younger patients or those needing repeated imaging. This makes MRI not only a powerful diagnostic tool but also a patient-friendly option for ongoing cancer care [52].

### 3.4. Magnetic Resonance Spectroscopy (MRS)

MRS provides a detailed analysis of the biochemical composition of tissues, offering metabolic information that enhances traditional anatomical imaging. When MRI is integrated with PET (PET-MRI), it combines the metabolic data from PET with the detailed soft tissue imaging from MRI, providing a thorough assessment of tumor characteristics. Advancements in CT, PET-CT, and MRI have greatly improved the ability to diagnose and manage NSCLC [53]. These imaging techniques offer complementary insights essential for accurate diagnosis, staging, and treatment planning. Innovations like low-dose protocols, advanced tracers, and hybrid imaging techniques promise to further enhance early detection and treatment of NSCLC, leading to better patient outcomes [54].

### 3.5. Molecular Diagnostic Biomarkers

#### 3.5.1. Genetic Profiling

NSCLC is marked by a variety of genetic mutations and alterations, such as EGFR, KRAS, ALK, ROS1, and BRAF. Identifying these mutations through genetic profiling allows for the development of targeted therapies tailored to the tumor’s specific molecular features. Next-generation sequencing (NGS) has emerged as a powerful tool for comprehensive genetic profiling, enabling the detection of multiple mutations simultaneously [55,56]. NGS provides detailed insights into the tumor’s genetic landscape, guiding personalized treatment strategies. Genetic profiling informs the use of targeted therapies, including EGFR inhibitors (erlotinib and gefitinib), ALK inhibitors (crizotinib and alectinib), and others. Additionally, it helps predict treatment response and potential resistance mechanisms, ensuring a more effective and personalized approach to managing NSCLC [56].

#### 3.5.2. Liquid Biopsies

Liquid biopsies analyze circulating tumor DNA (ctDNA) in the blood, offering a non-invasive way to profile tumors. They detect genetic mutations, monitor treatment response, and identify resistance mutations. Liquid biopsies provide real-time insights into tumor dynamics, enabling early relapse detection and treatment adjustments [57]. They are especially useful for patients who cannot undergo tissue biopsies or when tumor heterogeneity is a concern. Minimally invasive and repeatable, liquid biopsies give a comprehensive view of the tumor’s genetic changes over time.

The importance of performing liquid biopsies and detecting circulating tumor cells (CTCs) in NSCLC is crucial for identifying activating genetic mutations, such as ALK (anaplastic lymphoma kinase) rearrangements. This non-invasive technique allows for the analysis of tumor-derived material in the blood, providing real-time insights into the tumor’s genetic profile. It enables early detection and continuous monitoring of ALK mutations, facilitating timely and targeted treatment. Liquid biopsy is less invasive than traditional tissue biopsies, reducing patient discomfort and risk while allowing for repeated testing. By capturing genetic information from various tumor sites, it addresses tumor heterogeneity and provides a comprehensive understanding of the cancer’s genetic landscape. Identifying ALK mutations through liquid biopsies supports personalized treatment with specific ALK inhibitors, significantly improving patient outcomes through tailored therapy plans.

#### 3.5.3. Integration of Imaging and Molecular Diagnostics

Combining advanced imaging techniques with molecular diagnostics significantly improves the diagnosis and treatment of NSCLC. This integrated approach offers a comprehensive view of the tumor’s anatomical, functional, and molecular characteristics [58]. Advanced imaging techniques, such as CT, PET-CT, and MRI, help identify suspicious lesions and assess their properties. Molecular diagnostics, including genetic profiling and liquid biopsies, further analyze these lesions at the genetic level, identifying actionable mutations and guiding targeted therapy decisions. This holistic method enhances diagnostic accuracy and personalizes treatment strategies, ensuring that therapies are precisely tailored to the tumor’s specific molecular profile [59]. Regular imaging and molecular monitoring enable timely assessment of treatment response and early detection of resistance, allowing for dynamic adaptation of treatment plans to improve clinical outcomes. The integration of advanced imaging techniques with molecular diagnostics marks a significant advancement in the diagnosis and management of NSCLC [60]. These innovations facilitate precise tumor characterization, early detection, and personalized treatment, ultimately enhancing patient outcomes. Ongoing research and technological advancements promise to further enhance these capabilities, offering new hope in the fight against NSCLC. The future of NSCLC diagnosis and treatment lies in the continued integration of advanced imaging and molecular diagnostics. Emerging technologies, such as artificial intelligence (AI) and machine learning, hold promise for improving image analysis, diagnostic accuracy, and treatment outcome predictions [61]. Additionally, advancements in molecular diagnostics, including new biomarkers and more sensitive liquid biopsy techniques, will continue to refine personalized treatment approaches.

## 4. Innovative Therapies in NSCLC

The treatment of NSCLC has seen remarkable progress over the past decade. Advances in surgical techniques, targeted therapies, immunotherapies, and supportive care protocols have all contributed to better patient outcomes. In particular, innovations in surgery have been crucial for improving the prognosis of patients with early-stage NSCLC [62]. This section highlights these therapeutic innovations, especially focusing on the latest surgical advances and minimally invasive techniques (Figure 3).

### 4.1. Surgical Advances in NSCLC

Surgery remains a vital part of treating early-stage NSCLC. Advances in surgical methods and perioperative care have significantly lowered complications and deaths, sped up recovery, and increased long-term survival rates. Key advancements in surgery include using minimally invasive techniques and improved recovery protocols [63].

#### 4.1.1. Minimally Invasive Techniques

Minimally invasive surgery for NSCLC has revolutionized the field, offering several advantages over traditional open thoracotomy, including reduced postoperative pain, shorter hospital stays, faster recovery, and improved cosmetic outcomes [64]. Two primary minimally invasive techniques are video-assisted thoracoscopic surgery (VATS) and robotic-assisted surgery.

##### Video-Assisted Thoracoscopic Surgery (VATS)

VATS utilizes a thoracoscope, a tiny camera, and specialized surgical tools inserted through small chest incisions. Surgeons view the surgical area on a screen, enhancing their precision during the procedure. VATS is commonly used for procedures like lobectomy (removal of a lung lobe), wedge resection (removal of a small section of lung tissue), and segmentectomy (removal of a lung segment). Compared to traditional open surgery, VATS results in less pain after surgery, shorter hospital stays, quicker recovery to normal activities, and fewer complications. It has become the preferred method for treating early-stage NSCLC [65]. The minimally invasive nature of VATS offers improved cosmetic outcomes due to smaller incisions and reduces the risk of postoperative infections and respiratory issues. The thoracoscope’s enhanced visualization helps surgeons make precise dissections, crucial for achieving clear surgical margins and complete tumor removal. Additionally, VATS enables thorough lymph node dissection, vital for accurate staging and determining suitable adjuvant therapies. As VATS techniques and tools continue to advance, its applications are expanding, making it feasible for more complex cases and advanced-stage disease [66].

##### Robotic-Assisted Surgery

Robotic-assisted surgery uses advanced technology like the da Vinci Surgical System, providing surgeons with a three-dimensional, high-definition view of the surgical area and highly precise instruments [67]. Like VATS, robotic surgery is utilized for procedures such as lobectomy, segmentectomy, and other lung resections. It offers improved visualization, increased precision, and enhanced control over surgical tools, potentially leading to better outcomes, particularly in complex cases requiring intricate dissection and reconstruction. Robotic surgery allows for smaller incisions, which can reduce postoperative pain and speed up recovery times. The precise control and stability of robotic instruments are especially beneficial in maneuvering around delicate structures and blood vessels, minimizing the risk of complications [68]. Additionally, robotic-assisted surgery aids in thorough lymph node dissection, crucial for accurate staging and prognosis in NSCLC. The enhanced dexterity and stability of robotic instruments also enable more refined suturing and tissue manipulation, making it a preferred option for challenging resections and reconstructions. As technology continues to progress, integrating robotic systems with augmented reality and real-time imaging could further transform the surgical approach to NSCLC, offering even more precise and effective treatments.

##### Enhanced Recovery after Surgery (ERAS) Protocols

ERAS protocols are structured plans based on research to improve surgical outcomes and speed up recovery. They cover actions before, during, and after surgery to lessen the body’s stress, lower complications, and promote faster healing [69].

##### Preoperative Measures

(i) Providing comprehensive preoperative education about the surgical procedure, recovery process, and expectations helps reduce anxiety and improve patient engagement; (ii) managing comorbidities such as diabetes, hypertension, and chronic obstructive pulmonary disease (COPD) preoperatively enhances surgical outcomes; and (iii) ensuring optimal nutritional status preoperatively supports immune function and wound healing.

##### Intraoperative Measures

(i) Utilizing VATS or robotic-assisted surgery minimizes tissue trauma and reduces postoperative pain (minimally invasive techniques); (ii) employing multimodal anesthesia, including regional blocks and local anesthesia, reduces the need for opioids and enhances postoperative recovery; and (iii) optimizing intraoperative fluid management prevents fluid overload and reduces the risk of complications such as pulmonary edema.

##### Postoperative Measures

(i) Encouraging early ambulation and physical activity helps prevent complications such as deep vein thrombosis (DVT) and promotes faster recovery; (ii) utilizing multimodal analgesia, including non-opioid medications, reduces pain and opioid-related side effects; (iii) implementing early oral feeding and maintaining adequate nutrition supports recovery and wound healing; and (iv) providing respiratory exercises and pulmonary rehabilitation reduces the risk of postoperative pulmonary complications and enhances lung function. A concise table (Table 1) highlighting the importance of selecting the appropriate surgical approach in NSCLC is given.

Recent advancements in radiotherapy have significantly improved the treatment outcomes for NSCLC. Two significant advancements are Stereotactic Body Radiotherapy (SBRT) and Intensity-Modulated Radiotherapy (IMRT). These methods, combined with better treatment planning, have resulted in a notable enhancement in treatment outcomes [70].

### 4.2. Stereotactic Body Radiotherapy (SBRT)

SBRT, also called stereotactic ablative radiotherapy (SABR), is an extremely precise type of radiation therapy that administers high radiation doses to small, well-defined tumors over a limited number of treatment sessions, usually one to five. It relies on advanced imaging methods and careful planning to precisely target the tumor while reducing exposure to healthy surrounding tissues [71]. SBRT employs advanced imaging technologies like CT, MRI, and PET scans to pinpoint the tumor accurately. This accuracy is further improved with the use of fiducial markers and respiratory gating to accommodate tumor movement caused by breathing. SBRT delivers a concentrated radiation dose per treatment, causing significant tumor cell death while protecting nearby normal tissues. This is particularly advantageous for early-stage NSCLC and patients who cannot undergo surgery for medical reasons. Research indicates that SBRT achieves similar local control rates as surgery for early-stage NSCLC, with side effects and quicker recovery times [72]. Benefits of SBRT include the ability to deliver high doses in fewer sessions, reducing overall treatment time and making it more convenient for patients. Additionally, SBRT’s precise targeting reduces the radiation dose to surrounding healthy tissues, minimizing side effects like radiation pneumonitis and esophagitis.

### 4.3. Intensity-Modulated Radiotherapy (IMRT)

IMRT is an advanced technique in radiotherapy that adjusts the intensity of radiation beams to precisely target tumors while protecting nearby healthy tissues. This is conducted through computer-guided algorithms that optimize the distribution of radiation based on the tumor’s size and position. IMRT employs multiple beams of varying intensities from different angles to shape the radiation dose according to the tumor’s three-dimensional structure [73]. Modern imaging and planning software allows for adaptive radiotherapy, which adapts treatment plans as the tumor changes in size or position during treatment. This targeted approach improves the control of the tumor and overall survival rates by delivering higher radiation doses to the tumor while reducing radiation exposure to surrounding organs like the lungs, heart, and esophagus [74]. IMRT is particularly effective for tumors in challenging locations and benefits from integrating advanced imaging techniques such as 4D-CT and MRI for precise tumor targeting, considering tumor motion and anatomical changes during treatment. IMRT offers benefits in terms of patient comfort and convenience [75]. IMRT’s capacity to administer increased doses over fewer sessions streamlines treatment, enhancing patient manageability. The pinpoint accuracy of IMRT translates to diminished side effects, notably radiation-induced pneumonitis, and esophagitis, culminating in an improved quality of life throughout and post-treatment.

### 4.4. Innovations in Treatment Planning

The advancements in radiotherapy for NSCLC are complemented by innovations in treatment planning, which ensure the optimal delivery of radiation doses while minimizing risks to healthy tissues [76].

#### 4.4.1. Advanced Imaging Techniques

##### Four-Dimensional CT Imaging

Four-dimensional CT imaging captures tumor motion due to respiration, allowing for more accurate targeting and dose delivery [77].

##### MRI Integration

The use of MRI in treatment planning provides superior soft tissue contrast, improving the delineation of tumor boundaries and surrounding structures [78].

#### 4.4.2. Adaptive Radiotherapy

##### Real-Time Monitoring

Techniques such as real-time tumor tracking and respiratory gating allow for adjustments during radiation delivery, accommodating for patient movement and tumor position changes.

##### Adaptive Replanning

Regular imaging during treatment allows for adaptive replanning, where the radiation plan is updated based on changes in tumor size and shape. This enhances the precision and effectiveness of the treatment. Advances in radiotherapy for NSCLC, particularly through SBRT and IMRT, have revolutionized disease management. These techniques offer precise targeting, high radiation doses, and reduced side effects, significantly improving patient outcomes. Innovations in treatment planning, such as advanced imaging and adaptive radiotherapy, further enhance the precision and effectiveness of radiotherapy, making it a key component of NSCLC’s multimodal treatment approach. Ongoing research and technological advancements promise to continue refining these techniques, offering hope for even better outcomes in the future [79]

## 5. Systemic Therapy Innovations in NSCLC

Systemic therapies have revolutionized the treatment of NSCLC by providing targeted and personalized approaches that greatly enhance patient outcomes. Major innovations in systemic therapy include targeted inhibitors for specific genetic mutations like EGFR, ALK, and ROS1 [80]. These therapies have transformed NSCLC treatment by offering highly effective, mutation-specific options that are generally less toxic than traditional chemotherapy.

### 5.1. Epidermal Growth Factor Receptor (EGFR) Inhibitors

Mutations in the EGFR gene are found in approximately 10–15% of NSCLC cases, particularly among non-smokers and patients of East Asian descent. EGFR mutations lead to the activation of downstream signaling pathways that promote tumor cell proliferation and survival.

#### 5.1.1. First-Generation EGFR Inhibitors (Examples: Erlotinib and Gefitinib)

These drugs are tyrosine kinase inhibitors (TKIs) that block the ATP-binding site of the EGFR tyrosine kinase domain, thus inhibiting its signaling activity. They have shown significant effectiveness in extending progression-free survival (PFS) compared to chemotherapy in patients with EGFR-mutant NSCLC. However, resistance often develops within a year due to secondary mutations like T^790^M [81].

#### 5.1.2. Second-Generation EGFR Inhibitors (Examples: Afatinib and Dacomitinib)

These inhibitors are designed to irreversibly bind to the EGFR tyrosine kinase domain, providing a more sustained inhibition. Second-generation inhibitors have demonstrated improved efficacy over first-generation TKIs and chemotherapy [82]. They offer prolonged PFS but are also associated with increased toxicity.

#### 5.1.3. Third-Generation EGFR Inhibitors (Example: Osimertinib)

Osimertinib selectively targets both the sensitizing EGFR mutations and the T^790^M resistance mutation. Osimertinib has shown superior efficacy in overcoming T^790^M-mediated resistance and has become a standard first-line treatment for patients with EGFR-mutant NSCLC. It offers improved progression-free survival (PFS) and overall survival (OS) with a favorable safety profile. In a significant clinical trial involving patients with advanced EGFR-mutated stage III NSCLC, the drug osimertinib markedly prolonged PFS compared to a placebo-controlled trial, with patients living without disease progression for an average of 39.1 months vs. just 5.6 months. After one year, 74% of those treated with osimertinib remained alive and free from disease progression, compared to only 22% of those on placebo. While serious side effects were more frequent with osimertinib (35% vs. 12%), no new safety concerns emerged [83].

### 5.2. Anaplastic Lymphoma Kinase (ALK) Inhibitors

Rearrangements in the ALK gene are present in about 5% of NSCLC cases, predominantly in younger patients and non-smokers. These rearrangements result in the production of a constitutively active ALK fusion protein that drives oncogenesis [84].

#### 5.2.1. First-Generation ALK Inhibitors (Example: Crizotinib)

Crizotinib inhibits ALK tyrosine kinase activity, blocking downstream signaling pathways involved in cell proliferation and survival. Crizotinib significantly improves PFS and response rates compared to chemotherapy in ALK-positive NSCLC. However, patients eventually develop resistance, often due to secondary mutations or the activation of bypass signaling pathways [85].

#### 5.2.2. Second-Generation ALK Inhibitors (Examples: Ceritinib, Alectinib, and Brigatinib)

These inhibitors are designed to overcome crizotinib resistance and have better central nervous system (CNS) penetration. Clinical Outcomes: Second-generation ALK inhibitors have shown superior efficacy over crizotinib in both crizotinib-naïve and resistant patients. They provide longer PFS and improved CNS control [86].

#### 5.2.3. Third-Generation ALK Inhibitor (Example: Lorlatinib)

Lorlatinib is a potent ALK inhibitor that targets a broad range of resistance mutations and has excellent CNS penetration. Lorlatinib is effective in patients who have developed resistance to second-generation inhibitors, offering improved PFS and CNS control [87].

### 5.3. ROS1 Inhibitors

ROS1 rearrangements occur in about 1–2% of NSCLC cases, leading to the expression of oncogenic ROS1 fusion proteins that drive tumor growth [88].

#### 5.3.1. Crizotinib

Crizotinib is a multi-targeted TKI that inhibits ROS1, ALK, and MET. Crizotinib has shown high response rates and prolonged PFS in ROS1-positive NSCLC. However, resistance mechanisms similar to those seen in ALK-positive cases can limit its long-term efficacy.

#### 5.3.2. Second-Generation ROS1 Inhibitors (Examples: Entrectinib and Lorlatinib)

These inhibitors are designed to overcome crizotinib resistance and have better CNS penetration. Entrectinib and lorlatinib have shown efficacy in ROS1-positive NSCLC patients, including those with CNS metastases, offering longer PFS and better overall control of the disease [89].

### 5.4. Other Targeted Inhibitors

#### 5.4.1. BRAF Inhibitors (Example: Dabrafenib (Often Combined with Trametinib))

Dabrafenib targets BRAF V^600^E mutations, while trametinib inhibits MEK, a downstream effector in the MAPK pathway. The combination of dabrafenib and trametinib has demonstrated significant efficacy in BRAF V^600^E-mutant NSCLC, providing improved response rates and PFS compared to chemotherapy [90].

#### 5.4.2. MET Inhibitors (Examples: Capmatinib and Tepotinib)

These inhibitors target MET exon 14 skipping mutations and MET amplifications, which are involved in tumor growth and survival. MET inhibitors have shown promising results in patients with MET exon 14-altered NSCLC, offering high response rates and durable responses [91].

#### 5.4.3. RET Inhibitors (Examples: Selpercatinib and Pralsetinib)

These inhibitors specifically target RET rearrangements, which drive oncogenesis in a subset of NSCLC patients. RET inhibitors have demonstrated high efficacy and good tolerability in RET-rearranged NSCLC, providing substantial clinical benefits [92].

### 5.5. Combination Therapies

Combining targeted therapies with other treatment modalities, such as immunotherapy and chemotherapy, has shown promising results in enhancing therapeutic efficacy and overcoming resistance mechanisms [93]. Some of these combinations include the following.

#### 5.5.1. Targeted Therapy and Immunotherapy

EGFR inhibitors combined with immune checkpoint inhibitors (e.g., pembrolizumab) are being explored to enhance antitumor immunity and improve outcomes in EGFR-mutant NSCLC. Early-phase trials indicate that combining targeted therapies with immunotherapy may provide synergistic effects, potentially leading to improved survival and response rates [94].

#### 5.5.2. Targeted Therapy and Chemotherapy

ALK inhibitors combined with chemotherapy have been studied to manage resistance and enhance therapeutic efficacy in ALK-positive NSCLC. Combination regimens have shown the potential to delay resistance and improve overall treatment outcomes, though careful management of toxicity is necessary [95].

#### 5.5.3. Precision Medicine and Future Directions

The continued integration of precision medicine into NSCLC treatment involves comprehensive genomic profiling and the development of next-generation sequencing technologies to identify actionable mutations and tailor therapies accordingly [96].

#### 5.5.4. Comprehensive Genomic Profiling

NGS panels can identify multiple genetic changes at once, including point mutations, insertions/deletions, copy number variations, and fusions. This comprehensive analysis provides a detailed view of the tumor’s molecular profile, helping doctors choose the best targeted therapies and combination treatments [97].

#### 5.5.5. Emerging Targets

New targets like HER2, NTRK, and KRAS G^12^C are under investigation, with promising inhibitors showing potential in clinical trials. These new therapies bring hope to patients with previously untreatable or resistant forms of NSCLC, expanding the range of targeted treatment options (Table 2). Innovations in systemic therapy, especially targeted inhibitors, have greatly advanced NSCLC treatment. Inhibitors targeting EGFR, ALK, ROS1, and other mutations offer personalized treatment options that significantly improve clinical outcomes, including longer progression-free survival and overall survival, with less toxicity than traditional chemotherapy [98]. These advancements highlight the importance of molecular profiling in NSCLC, enabling the selection of the most appropriate targeted treatments for each patient. Ongoing research and the development of next-generation inhibitors continue to expand treatment options, promising even better outcomes for NSCLC patients in the future. Integrating precision medicine approaches and combination therapies holds great potential to further enhance NSCLC treatment effectiveness and tackle challenges like resistance and disease variability [99].

## 6. Challenges in NSCLC Management

Managing NSCLC presents a variety of challenges that impact both the efficacy of treatments and the equity of care provided to patients. These challenges can be broadly categorized into healthcare disparities, economic, and ethical issues [100].

### 6.1. Healthcare Disparities (Access to Diagnostic and Therapeutic Advances)

A significant hurdle in dealing with NSCLC is the uneven availability of advanced diagnostic and treatment methods. Individuals from less affluent backgrounds, rural locales, or minority communities frequently encounter notable obstacles in receiving timely and suitable care. These barriers encompass restricted entry to top-notch healthcare facilities, a deficit of specialists, and financial limitations [101]. Sophisticated diagnostic instruments such as 4D-CT and integrated MRI are frequently absent in numerous healthcare setups, particularly in regions with limited resources. This absence can result in delayed diagnoses, incomplete staging, and less precise treatment plans, ultimately impacting patient outcomes negatively. Likewise, cutting-edge treatments like targeted therapies and immunotherapies are often inaccessible to numerous patients due to their steep prices and restricted availability in specific regions. These healthcare inequalities are aggravated by variances in health literacy and patient education, influencing a patient’s capacity to seek medical attention, comprehend treatment choices, and adhere to prescribed regimens [102]. Tackling these disparities necessitates endeavors to enhance healthcare infrastructure, offer financial aid, and establish policies guaranteeing fair access to advanced medical technologies and treatments for every patient.

### 6.2. Economic and Ethical Issues (Cost-Effectiveness and Ethical Considerations in Treatment)

The financial strain of treating NSCLC presents considerable hurdles for both patients and healthcare systems. The steep expenses tied to advanced diagnostic imaging, targeted therapies, and immunotherapies can be daunting, compelling many patients to navigate tough decisions about their treatment journey [103]. These financial limitations frequently lead patients and their families to opt for less effective treatments due to financial constraints. Assessing the cost-effectiveness of NSCLC treatments becomes imperative. Although these advanced therapies offer significant clinical benefits, their hefty price tags raise concerns about their overall worth and long-term viability. Health economists and policymakers must scrutinize the cost-effectiveness of these treatments to ensure they deliver adequate value for the investment made. This evaluation requires a delicate balance between the costs incurred and the benefits derived, considering factors such as survival rates, quality of life, and enduring health outcomes [104]. Ethical considerations are paramount in NSCLC management. Guaranteeing equal access to life-saving treatments for all patients, irrespective of their socio-economic status, poses a significant ethical dilemma. There is an ethical obligation to offer optimal care to everyone, yet financial constraints and limited resources often necessitate difficult prioritization decisions [105]. Moreover, the ethical ramifications of emerging technologies and treatments, such as incorporating artificial intelligence in treatment planning or utilizing genetic profiling for personalized medicine, demand careful assessment. Issues like patient consent, privacy, and the potential for genetic information-based discrimination must be guided by robust ethical frameworks to steer clinical practices and policies effectively [104,105,106].

## 7. Emerging Research and Future Prospects

The future of NSCLC management looks bright, with many innovative therapies and groundbreaking research underway (Table 3 and Table 4). These advancements have the potential to revolutionize NSCLC treatment, leading to better patient outcomes and providing new hope for those battling this difficult disease [107].

### 7.1. Cutting-Edge Research and Experimental Therapies

#### 7.1.1. CAR-T Cell Therapy

Chimeric antigen receptor T-cell (CAR-T) therapy, a groundbreaking method in cancer treatment, involves engineering T cells with receptors designed to target and eliminate tumor cells. In NSCLC, scientists are actively investigating potential targets like mesothelin, EGFR, and PD-L1 for CAR-T therapy. Initial clinical trials are underway to evaluate the safety and effectiveness of CAR-T cells in NSCLC patients, showing encouraging early outcomes. This innovative approach holds promise for providing long-lasting remissions, especially for those with challenging-to-treat or advanced-stage disease [108]. The ongoing research and development in CAR-T therapy signifies a hopeful direction in the quest for more effective treatments against NSCLC.

#### 7.1.2. Oncolytic Viruses

Oncolytic viruses are a sophisticated form of treatment engineered to infect and destroy cancer cells while leaving healthy cells unharmed. They not only directly attack tumors but also trigger the immune system’s response against cancer. In NSCLC, various oncolytic viruses like talimogene laherparepvec (T-VEC) and adenoviruses are currently undergoing clinical trials to evaluate their effectiveness. These viruses can be customized to carry therapeutic genes, such as immune checkpoint inhibitors or cytokines, amplifying their ability to combat cancer. Combining oncolytic virotherapy with other immunotherapies shows promise as a cutting-edge treatment approach for NSCLC, potentially revolutionizing how we combat this disease [109].

### 7.2. Promising Clinical Trials and Their Potential Impact

#### 7.2.1. Immunotherapy Combinations

Studies investigating combinations of immunotherapies, such as checkpoint inhibitors like pembrolizumab and nivolumab, with other substances like CTLA-4 inhibitors, are demonstrating substantial promise in NSCLC. These combined treatments are designed to bolster the immune system’s response to tumors, resulting in better survival rates and treatment responses [110]. Researchers are also looking into using immunotherapies in earlier stages of NSCLC, both before and after surgery, which could potentially prevent cancer from returning and lead to better long-term results.

#### 7.2.2. Targeted Therapy Advances

Novel targeted treatments are continually being developed, honing in on fresh genetic and molecular changes seen in NSCLC. For instance, the latest EGFR inhibitors (like osimertinib) and ALK inhibitors (such as brigatinib and lorlatinib) are undergoing clinical trials to assess their ability to overcome resistance seen with earlier therapies [111]. Moreover, treatments aimed at rarer mutations, like KRAS G^12^C inhibitors (like sotorasib) and MET exon 14 skipping mutations, are showing encouraging results. These trials are essential for broadening the range of treatment choices for patients with specific genetic characteristics [112].

### 7.3. Future Directions in NSCLC Research and Treatment Development

#### 7.3.1. Personalized Medicine

The future of treating NSCLC revolves around personalized medicine, crafting therapies that suit the unique genetic and molecular makeup of each patient’s tumor. Innovations in genomic profiling and liquid biopsy techniques are making it possible to pinpoint actionable mutations and biomarkers with greater accuracy [113]. This method enables doctors to choose the most suitable treatments, reducing unnecessary therapies and their side effects. Current research is dedicated to integrating extensive genomic information into clinical decisions, paving the way for more individualized and successful treatment plans.

#### 7.3.2. Artificial Intelligence (AI) and Machine Learning

AI and machine learning stand on the brink of transforming NSCLC research and treatment. These cutting-edge technologies excel at processing massive datasets, recognizing patterns, and making highly accurate predictions. In NSCLC, AI plays a role in aiding diagnosis, staging, and predicting how patients respond to different treatments [114]. Meanwhile, machine learning algorithms are in the works to refine treatment planning, enhancing the precision of radiotherapy and the choice of targeted therapies. Incorporating AI into clinical settings has the potential to boost diagnostic precision, streamline treatment processes, and ultimately lead to better patient outcomes [115].

#### 7.3.3. Novel Therapeutic Targets

Research efforts are constantly unveiling fresh therapeutic possibilities in NSCLC. Emerging fields of exploration encompass honing in on the tumor’s microenvironment, cancer stem cells, and metabolic pathways. Investigations are underway for therapies that alter the immune microenvironment, like agents that target macrophages and T-cell engagers [116]. Moreover, delving into the metabolic needs of cancer cells, such as using glutaminase inhibitors, presents an intriguing avenue in research. These innovative strategies hold promise in tackling resistance mechanisms and opening up new paths for treatment [117].

## 8. Conclusions

The landscape of treating NSCLC has seen remarkable advancements thanks to breakthroughs like targeted therapies, immunotherapy, advanced imaging methods, minimally invasive surgeries, and radiotherapy developments. These innovations have brought about substantial improvements in patient well-being by providing accurate, efficient treatments with minimized side effects [118]. Nevertheless, hurdles persist, including disparities in healthcare that hinder access to these innovations, economic and ethical concerns due to treatment costs, the emergence of resistance to therapies, and the intricacies of tumor biology [119]. Looking ahead, the future of managing NSCLC holds the promise of further transformation through personalized medicine, the integration of AI and machine learning, the development of new therapies, and the exploration of combination treatments [120]. It is crucial to address healthcare disparities and ensure fair access to advanced treatments. Prioritizing patient-centered care and fostering global collaboration will play a pivotal role in advancing NSCLC treatment and enhancing patient outcomes. By continuously pushing the boundaries of research and ensuring that innovations benefit all patients, the medical community can make significant strides in improving survival rates and enhancing the quality of life for individuals impacted by NSCLC [121].

## 9. Clinical Impact of Advances in NSCLC

Advancements in NSCLC treatment have radically improved patient outcomes, offering new hope through personalized therapies. Molecular targeted therapies and immunotherapies have increased survival rates and quality of life, while innovations in diagnostic imaging and minimally invasive techniques ensure precise, early interventions with fewer side effects. These breakthroughs allow for tailored treatments, effectively addressing resistant and previously untreatable forms of NSCLC, indicating a transformative era in lung cancer care [122,123,124].

## 10. Significance of Advancement in NSCLC

(1) Personalized therapies, such as molecular targeted treatments and immunotherapies, have significantly improved survival rates and quality of life for NSCLC patients by providing effective treatments with fewer side effects.

(2) Advanced diagnostic imaging and minimally invasive techniques enable early, precise detection and treatment planning, while new therapies effectively address resistance and previously untreatable forms of NSCLC, revolutionizing patient care.

In summary, advances in NSCLC, including personalized therapies, advanced diagnostic imaging, and innovative treatment techniques, have significantly improved patient survival and quality of life, while ongoing research promises even greater future breakthroughs.

## Figures and Tables

**Table 1:** Different surgical approaches in NSCLC: descriptions, benefits, and potential complications, highlighting the importance of selecting the right approach based on patient characteristics and tumor factors.

Surgical Approach	Description	Benefits	Complications
Minimally Invasive Techniques	Utilizes VATS or robotic-assisted surgery, reducing trauma and enhancing precision.	Reduced postoperative pain, shorter hospital stays, faster recovery, improved cosmetic outcomes.	Risk of air leaks, prolonged chest tube duration, potential for incomplete tumor removal in complex cases.
Traditional Open Thoracotomy	Involves a larger chest incision for direct access, suitable for extensive or complex cases.	Allows for thorough tumor removal, suitable for larger tumors or complex anatomical situations.	Higher risk of postoperative pain, longer hospital stays, increased infection rates, longer recovery periods.
Specific Considerations	Selection based on tumor size, location, and patient factors.	Provides tailored treatment options, balances tumor removal with preservation of lung function.	Risk of complications varies based on the extent of resection (e.g., pneumonectomy vs. lobectomy vs. segmentectomy).

**Table 2:** Key innovations in the management of NSCLC, highlighting advancements in diagnosis, treatment, and patient care.

Advancement	Overview	Advantages	Examples
Molecular targeted therapies	Target specific genetic alterations (e.g., EGFR, ALK, and KRAS) using tyrosine kinase inhibitors (TKIs).	Improved survival, quality of life, and disease management.	Erlotinib, Crizotinib, Sotorasib
Immunotherapy	Use of immune checkpoint inhibitors (e.g., pembrolizumab and nivolumab) to enhance immune response.	Durable responses, potential for long-term remission.	Pembrolizumab, Nivolumab
Advanced imaging techniques	4D-CT and MRI integration for precise diagnosis and treatment planning.	Better tumor delineation, accurate targeting, improved monitoring.	4D-CT, MRI
Minimally invasive surgery	VATS and robotic-assisted surgery for lung resections.	Reduced pain, shorter hospital stays, faster recovery.	VATS, Da Vinci Surgical System
Radiotherapy advances	SBRT and IMRT for precise, high-dose radiation therapy.	High precision, minimized side effects, improved outcomes.	SBRT, IMRT

**Table 3:** Promising future directions in the management of NSCLC.

Aspect	Details	Current Limitations	Prospective Developments
Personalized medicine	Genomic profiling and liquid biopsy technologies for tailored treatment plans.	Limited availability and high costs.	Broader implementation and cost reduction through technological advances.
Integration of artificial intelligence	AI and machine learning for optimizing diagnosis, staging, and treatment plans.	Requires extensive data and validation.	Enhanced data collection, validation, and integration into clinical workflows.
Development of novel therapies	Emerging therapies like CAR-T cells, oncolytic viruses, and next-generation inhibitors.	Early stages of research, high costs, and potential side effects.	Extensive clinical trials, cost-effective production, and refinement of therapies.
Combination therapies	Combining modalities such as targeted therapies with immunotherapies or radiation with systemic treatments.	Potential for increased toxicity and complex treatment regimens.	Optimizing combinations and dosing to minimize toxicity while maximizing efficacy.
Global collaboration and equity	Efforts to improve access to advanced diagnostics and therapies worldwide, addressing healthcare disparities.	Variability in healthcare infrastructure and funding across regions.	International partnerships and funding initiatives to standardize access globally.
Patient-centered care	Emphasizing quality of life, supportive care measures, and incorporating patient preferences in treatment.	Requires comprehensive care models and integration of multidisciplinary teams.	Development of integrated care models that prioritize patient experience and outcomes.

**Table 4:** Persistent challenges in the management of NSCLC.

Challenges	Description	Current Limitations	Future Directions
Healthcare disparities	Limited access to advanced diagnostic tools and therapies in lower socioeconomic and rural areas.	Inconsistent healthcare policies and lack of infrastructure.	Policy reforms and investment in healthcare infrastructure to ensure equitable access.
Economic and ethical issues	High cost of treatments posing economic burdens and ethical dilemmas in equitable access.	Need for policy reforms and financial support mechanisms.	Implementation of cost-effective strategies and ethical guidelines for treatment access.
Resistance mechanisms	Development of resistance to targeted therapies and immunotherapies limiting long-term effectiveness.	Ongoing research required to understand and overcome resistance.	Research into new targets and combination strategies to overcome resistance.
Complex tumor biology	Heterogeneity of NSCLC complicating treatment due to diverse molecular alterations.	Personalized approaches needed for effective treatment of diverse tumor profiles.	Advancements in molecular profiling and personalized treatment plans.

## Data Availability

No new data were created or analyzed in this study. Data sharing is not applicable to this article.

## Abbreviations

AI: artificial intelligence; ALK: anaplastic lymphoma kinase; CAR-T: chimeric antigen receptor T cell; CT: computed tomography; CTLA-4: cytotoxic T-lymphocyte-associated protein 4; EGFR: epidermal growth factor receptor; ERAS: enhanced recovery after surgery; 4D-CT: four-dimensional computed tomography; HER2: human epidermal growth factor receptor 2; IMRT: intensity-modulated radiotherapy; KRAS: Kirsten rat sarcoma viral oncogene homolog; MET: mesenchymal-epithelial transition factor; MRI: magnetic resonance imaging; NGS: next-generation sequencing; NSCLC: non-small cell lung cancer; PD-L1: programmed death-ligand 1; PFS: progression-free survival; ROS1: c-ros oncogene 1; SBRT: stereotactic body radiotherapy; T-VEC: talimogene laherparepvec; TKI: tyrosine kinase inhibitor; VATS: video-assisted thoracoscopic surgery.
